# Antimicrobial strategy for targeted elimination of different microbes, including bacterial, fungal and viral pathogens

**DOI:** 10.1038/s42003-022-03586-4

**Published:** 2022-07-04

**Authors:** Makoto Mitsunaga, Kimihiro Ito, Takashi Nishimura, Hironori Miyata, Kei Miyakawa, Takeshi Morita, Akihide Ryo, Hisataka Kobayashi, Yoshimitsu Mizunoe, Tadayuki Iwase

**Affiliations:** 1 Division of Gastroenterology and Hepatology, Department of Internal Medicine, The Jikei University School of Medicine, Tokyo, Japan; 2 Animal Research Center, School of Medicine, University of Occupational and Environmental Health, Kitakyushu, Japan; 3 Department of Microbiology, Yokohama City University School of Medicine, Kanagawa, Japan; 4 Molecular Imaging Branch, Center for Cancer Research, National Cancer Institute, NIH, Bethesda, MD USA; 5 The Jikei University School of Medicine, Tokyo, Japan; 6 Research Center for Medical Sciences, The Jikei University School of Medicine, Tokyo, Japan

**Keywords:** Antibiotics, Molecular medicine, Drug development

## Abstract

The continuous emergence of microbial pathogens for which there are no effective antimicrobials threatens global health, necessitating novel antimicrobial approaches. Here, we present a targeted antimicrobial strategy that can be applied to various microbial pathogens. A photoimmuno-conjugate composed of an antibody against the target pathogen and a photoplastic phthalocyanine-derivative probe that generates photo-induced mechanical stress was developed based on photoimmuno-technology. This strategy, named as photoimmuno-antimicrobial strategy (PIAS), eliminates targeted pathogens, regardless of the target species or drug-resistance status. Specifically, PIAS acts on a broad range of microbes, including the bacterial pathogen *Staphylococcus aureus*, fungal pathogen *Candida albicans*, including their drug-resistant strains, and viral pathogen SARS-CoV-2, the causative agent of COVID-19. Furthermore, PIAS protects mice from fatal infections without damaging the non-targeted host microbiota and tissues. This study may contribute to the development of next-generation anti-infective therapies.

## Abstract (web summary)

The PIAS system, consisting of light-sensitive dye conjugated with monoclonal antibodies, is capable of eliminating fungal, viral, and bacterial pathogens, regardless of their drug resistance status.

## Introduction

Many antimicrobials have been developed (Table 1)^1^; however, microbes have rapidly developed resistance to newly developed drugs^2^, complicating infection treatment. Multidrug-resistant organisms are emerging rapidly with the increasing and indiscriminate use of antibiotics. Indeed, an estimated 4.95 million deaths were associated with bacterial antimicrobial resistance in 2019, making it a leading cause of death worldwide^3^. The bacterial pathogen *Staphylococcus aureus* (SA), particularly, methicillin-resistant SA (MRSA), is a notorious pathogen displaying multidrug resistance. SA is capable of colonising approximately 30% of the human nasal cavity asymptomatically^4,5^; however, MRSA is a causative agent of difficult-to-treat hospital-acquired infections^6^. Fungi with multidrug resistance, including the fungal pathogen *Candida albicans* (CA), are also involved in hard-to-treat infections. The biological and physiological characteristics of fungal cells, which are eukaryotic, closely resemble those of mammalian cells^7^; therefore, the development of fungicidal agents with selective toxicity towards fungal cells but not towards human cells remains challenging^7^.

**Table 1:** Antimicrobial strategies and target microbes and molecules.

Microbe (target species in this study)	Bacteria (*Staphylococcus aureus*, SA)	Fungi (*Candida albicans*, CA)	Viruses (SARS-CoV-2, SC; T7 phage)	
Description of biological differences	Bacteria based on the prokaryotic cells	Eukaryote based on the eukaryotic cells	Non-cellular organisms	Description of system
Antimicrobial strategy				
PIAS and its molecular targets/emergence of recalcitrant strains and their mechanisms (this study)	PIAS damages cell wall peptidoglycan of SA, including that of MRSA/n.d.	PIAS damages cell wall β-(1,2) mannan of CA, including that of drug-resistant strains/n.a.	PIAS damages a spike epitope of SC/n.a.; PIAS damages a capsid epitope of T7 phage/n.a.	System that uses anti-target pathogen antibody–photoplastic probe conjugates, enabling targeted elimination of different microbial pathogens, irrespective of their species or drug-resistant status
Conventional antimicrobials (antibiotics, antimycotics, and antiviral agents) and their molecular targets/drug-resistant strains and their mechanisms	β-Lactams and vancomycin inhibit bacterial cell wall peptidoglycan synthesis/drug degradation or alternative drug-targets show less affinity	Echinocandins inhibit fungal cell wall β-(1,3) D-glucan synthesis/mutated enzymes that show less affinity to the drugs	Remdesivir and favipiravir are clinically used against SC/n.a.; there is no agent against phage/n.a.	Systems that use antibiotics for bacteria, antimycotics for fungi, and antiviral agents, showing no effects on their drug-resistant strains (mainly antibiotics work against their biological group in addition to the target pathogen based on their characteristics, which can induce secondary infections via microbial substitutions following disruption of the normal microbial flora)
	Fluoroquinolones inhibit bacterial DNA synthesis/mutated target shows less affinity for the drugs	Azoles inhibit fungal cell membrane ergosterol synthesis/mutated enzymes show less affinity to the drugs		
	Macrolides, aminoglycosides, and linezolid inhibit bacterial protein synthesis/drug modification and mutated targets	Polyenes disrupt the fungal cell membrane/mutated enzymes show less affinity to the drugs		
	Daptomycin disrupts the bacterial cell membrane/n.a.	Fluorocytosine inhibits fungal DNA synthesis/mutated enzymes show less affinity to the drug		

*n.d.* not detected, *n.a*. data not available or undetermined.

Furthermore, the continuous evolution of new microbial pathogens in the absence of effective antimicrobials has become a major threat to global health. SARS-CoV-2 (SC), the causative agent of coronavirus disease (COVID-19), emerged in 2019^8^. SC immediately spread worldwide because of the lack of effective antiviral drugs and treatment approaches. Even after vaccines against SC were developed in 2021, casualties from COVID-19 have been increasing to date. As of April 1, 2022, there have been 486,761,597 confirmed cases of COVID-19, including 6,142,735 deaths, according to the WHO COVID-19 Dashboard (https://covid19.who.int/).

Given the current situation, effective countermeasures, including novel antimicrobial approaches, are urgently needed^1,2^. However, drug development generally takes more than 10 years, and almost all candidates are discarded in the early stages because of their toxicity^1^. In addition, the use of antibiotics can cause secondary infections via microbial substitutions by disrupting the normal microbial flora^9^. Furthermore, developed drugs often lose their effectiveness following the emergence of antimicrobial resistance^2^. For the development of future antimicrobial strategies, targeted antimicrobial approaches are ideal for avoiding the disruption of the normal microbial flora. More ideally, these approaches enable the targeted elimination of different pathogens, regardless of the target drug-resistant status. However, such antimicrobial approaches have not been developed thus far.

**Fig. 1:**
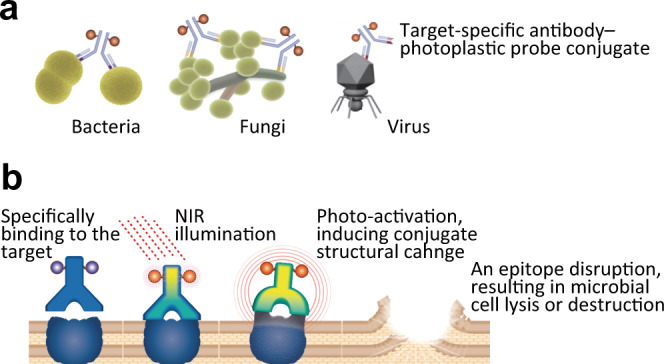
Target microbes and the proposed mechanism of PIAS. **a** Anti-target pathogen antibody–photoplastic probe IR700 conjugates bind to the target pathogens. **b** Scheme indicates the proposed microbicidal mechanism of PIAS based on our results.

To overcome these challenges, studies with interdisciplinary perspectives should be performed^10^, although it is generally challenging to find latent links among various fields of research. In 2011, we developed photoimmunotherapy, a novel anticancer therapy^11^ that uses a photoplastic phthalocyanine-derivative IRDye700DX (IR700) conjugated with monoclonal antibodies (mAb) targeting cancer cell membrane antigens, mAb panitumumab targeting human epidermal growth factor receptor (EGFR) and mAb trastuzumab^12^ targeting EGFR2 (HER2). Recent studies have proposed that the photo-activated probe induced a structural change of the probe and conjugate and generated mechanical stress that damaged the binding sites^11,13^. We predicted that this anticancer therapy could be extended to new applications, namely as an antimicrobial strategy targeting a broad range of microbes, including bacterial, fungal, and viral pathogens, regardless of their species or drug-resistance status (Fig. 1).

In this study, we developed a new type of targeted antimicrobial strategy, the photoimmuno-antimicrobial strategy (PIAS). PIAS is a targeted antimicrobial system applicable to different microbial pathogens, regardless of target drug-resistance status (Table 1).

## Results

### Bactericidal effect of PIAS

**Fig. 2:**
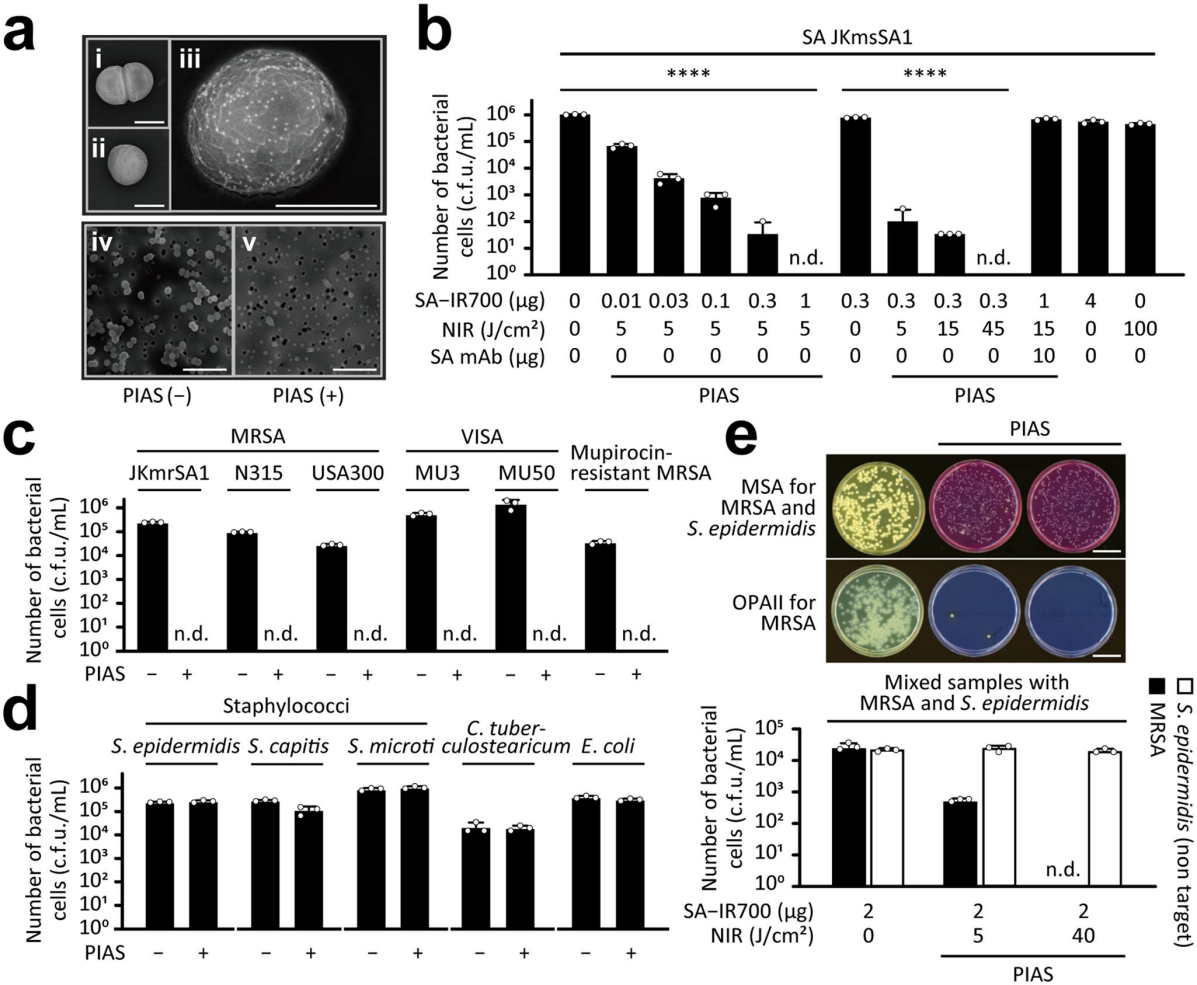
Bactericidal effect of PIAS. **a** Scanning electron microscopic (SEM) analysis of *Staphylococcus aureus* (SA) cells treated with SA–IR700 conjugate after immunogold staining (i–iii). Untreated (i) and colloidal gold IgG-treated cells (ii). (iii) Bright dots indicate the presence of conjugates. SEM analysis of untreated (iv) and PIAS-treated (v) SA cells. Scale bars indicate 0.5 µm (i–iii) and 5 µm (iv and v). **b**, **c** Bactericidal effect of PIAS on **b** SA and **c** drug-resistant SA strains. **d** Effect of PIAS on non-target bacteria. SA–IR700, 1 μg/test; near-infra-red light (NIR) illumination, 30 J/cm^2^. SA–IR700 was used in both PIAS (−) and (+) groups. **e** Target-selective bactericidal effect of PIAS. MRSA and *Staphylococcus epidermidis* formed yellow and pink colonies, respectively. Scale bars indicate 3 cm in (**e**). MSA mannitol salt agar with egg yolk, OPAII OPAII *Staphylococcus* agar, c.f.u. colony-forming units, n.d. not detected, n.s. not significant (*p* > 0.05). Mean and s.d. values are shown from triplicate samples; data and images are representative of at least three independent experiments. For statistical analyses, ANOVA (**b**) and ANOVA with the Sidak test (**e**) were performed. ****p < 0.0001.

Our first PIAS target was the bacterial pathogen SA, including MRSA. To develop PIAS against SA using a mAb against the SA-cell wall peptidoglycan epitope (SA mAb), we generated an SA-targeting conjugate (SA mAb–IR700 probe conjugate; SA–IR700). Target-selective binding of the conjugate was confirmed using scanning electron microscopy, fluorescence microscopy, and flow cytometry (i–iii, Fig. 2a and Supplementary Figs. 1–3). Using this conjugate, PIAS was then applied to SA. Briefly, the pathogen harvested from the stationary growth phase was treated with the conjugate SA–IR700 and then with NIR stimulation via a light-emitting diode releasing light at 670–710 nm NIR penetration into human deep tissues without side effects. The bactericidal effect of the treatment was determined using the colony counting method.

The bactericidal activity of PIAS against SA (10^5^ c.f.u./mL) was conjugate- and NIR-dose-dependent and occurred within several minutes of NIR illumination (5 J/cm^2^, 1 min) (Fig. 2b). SA at concentrations of both 10^7^ and 10^8^ c.f.u./mL was treated with PIAS, resulting in the elimination of both SA samples (Supplementary Fig. 4). The bactericidal effect of PIAS was observed in SA cells in both the exponential (Supplementary Fig. 5a) and stationary (Fig. 2b) phases. PIAS also acted on the SA cells under various conditions, including different culture media and conditions (Supplementary Fig. 5b–d). PIAS eliminated approximately 90% of SA cells in the biofilms (Supplementary Fig. 6). PIAS, with an SA mAb targeting different epitopes, also exerted this bactericidal effect (Supplementary Fig. 7). In addition to MRSA, PIAS eliminated various other drug-resistant SA strains (Fig. 2c and Supplementary Fig. 8); among the different drug-resistant strains tested, there was no difference in their response to PIAS.

In contrast, PIAS using a non-specific conjugate (HER2-targeting trastuzumab–IR700, Tra–IR700) had no significant effect on SA-deficient protein A, which binds to IgG through the Fc region of the antibodies (Supplementary Fig. 9). This suggests that non-specific binding through the Fc region of antibodies did not substantially contribute to antimicrobial activity (Fig. 1). We also investigated the contribution of oxygen toxicity typically observed in photodynamic therapy^14^ using ascorbate and catalase; the former eliminates oxidants^15,16^, such as singlet oxygen, and the latter degrades hydrogen peroxide. The experiment revealed that 100% of SA cells (~10^6^ c.f.u./mL) were eliminated by PIAS (Supplementary Fig. 10). However, 99.9% of SA cells (~10^4^ c.f.u. of 10^6^ c.f.u./mL) were eliminated in the presence of 1 mM ascorbate, but not in 200 U catalase (Supplementary Fig. 10). These results suggest the involvement of singlet oxygen, but not hydrogen peroxide, in the effects of PIAS. Additionally, PIAS using non-specific conjugates had no significant effects on fibroblasts in the presence of unbound free conjugates, which are more sensitive to oxygen toxicity than bacteria (Supplementary Fig. 11). Collectively, these results suggest that oxygen toxicity and non-specific conjugation did not contribute to the bactericidal effect of PIAS on the target pathogen.

We also investigated the effect of PIAS on non-target microbes using the normal host microflora, including the human commensal bacterium *Staphylococcus epidermidis* and animal commensal *Staphylococcus microti*. In these bacteria, no apparent effects were observed with PIAS using the SA-targeting conjugate (Fig. 2d). Furthermore, PIAS against SA selectively eliminated the target pathogen MRSA within mixed samples of MRSA and non-target *S*. *epidermidis* (Fig. 2e). These results indicate that PIAS with the SA-targeting conjugate specifically targets SA. In addition, flow cytometric analysis revealed that the SA mAb conjugate binding to *S*. *epidermidis* was much weaker than the binding to SA (Supplementary Fig. 12). PIAS with SA–IR700 had no apparent effect on *S*. *epidermidis* (Supplementary Fig. 12 and Fig. 2d, e). This discrimination effect of PIAS using SA-targeting conjugates on SA and *S*. *epidermidis* was considered to result from the antibody specificity. Together, PIAS showed targeted elimination of microbes.

Furthermore, SEM analysis revealed nearly no bacterial cell debris in the PIAS-treated SA samples (iv and v, Fig. 2a). The bactericidal effect of PIAS appears to be mechanical because of its rapid action, supporting the proposed mechanism of photoimmuno-technology on cancer cells^11,13^ in previous studies. In this context, strains recalcitrant to PIAS are unlikely to emerge; indeed, during the study period, recalcitrant strains were not observed (Supplementary Fig. 13).

### Fungicidal effect of PIAS

**Fig. 3:**
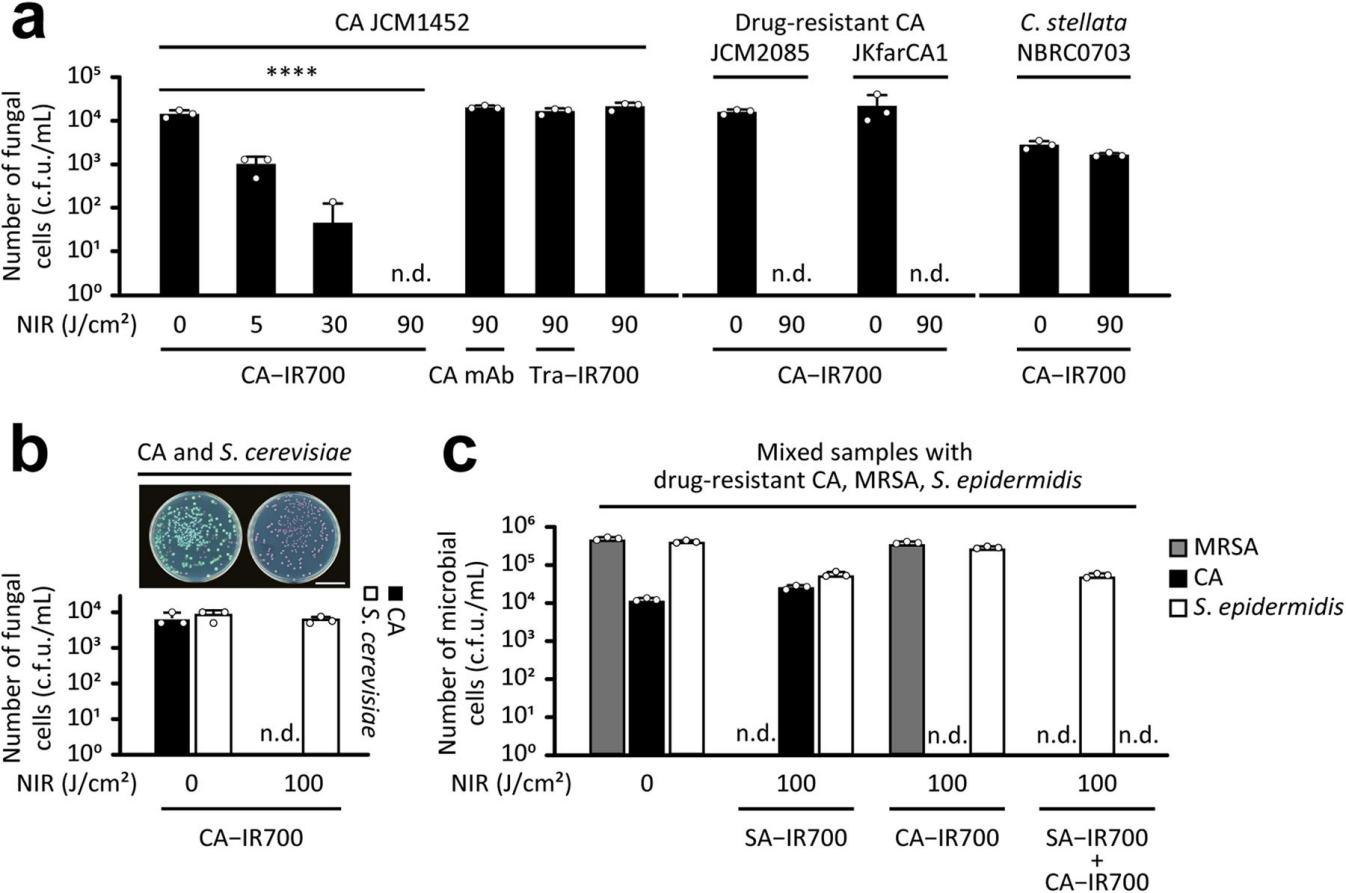
Fungicidal effect of PIAS. **a**, **b** Effects of PIAS on **a** *Candida albicans* (CA), drug-resistant CA strains, and the non-target/non-pathogenic fungi *Candida stellata* and on **b** *Saccharomyces cerevisiae*. The scale bar indicates 3 cm in (**b**). **c** Target-selective microbicidal effect of PIAS using CA–IR700 and/or SA–IR700 conjugate(s) on a mixed sample of drug-resistant CA, MRSA, and the non-target/commensal bacterium *Staphylococcus epidermidis*. CA and *S*. *cerevisiae* formed turquoise-blue and mauve colonies, respectively. CA–IR700, 20 μg/test; SA–IR700, 1 μg/test; NIR near-infra-red light, c.f.u. colony-forming units, n.d. not detected, n.s. not significant (*p* > 0.05). Mean and s.d. values are shown for triplicate samples; data were representative of at least three independent experiments. For statistical analyses, ANOVA with Dunnett’s test was performed. ****p < 0.0001.

Next, to further investigate the application of PIAS to a broad range of microbial pathogens, we targeted the fungal pathogen CA. To develop a CA-targeting PIAS, we generated a conjugate using a mAb against a CA-cell wall mannan epitope. PIAS using the CA–IR700 conjugate specifically showed a fungicidal activity, regardless of its drug-resistance status (Fig. 3a), but not non-targeted fungi, such as *Candida stellata* and *Saccharomyces cerevisiae* (Fig. 3a, b). Moreover, PIAS with CA–IR700 or SA–IR700 specifically eliminated CA or MRSA, whereas PIAS using both CA–IR700 and SA–IR700 eliminated both CA and MRSA, respectively (Fig. 3c). In these cases, non-target *S*. *epidermidis* was not eradicated (Fig. 3c). Together, PIAS using multiple conjugates selectively eliminates multiple targeted pathogens without affecting non-targets.

### Virucidal effect of PIAS

**Fig. 4:**
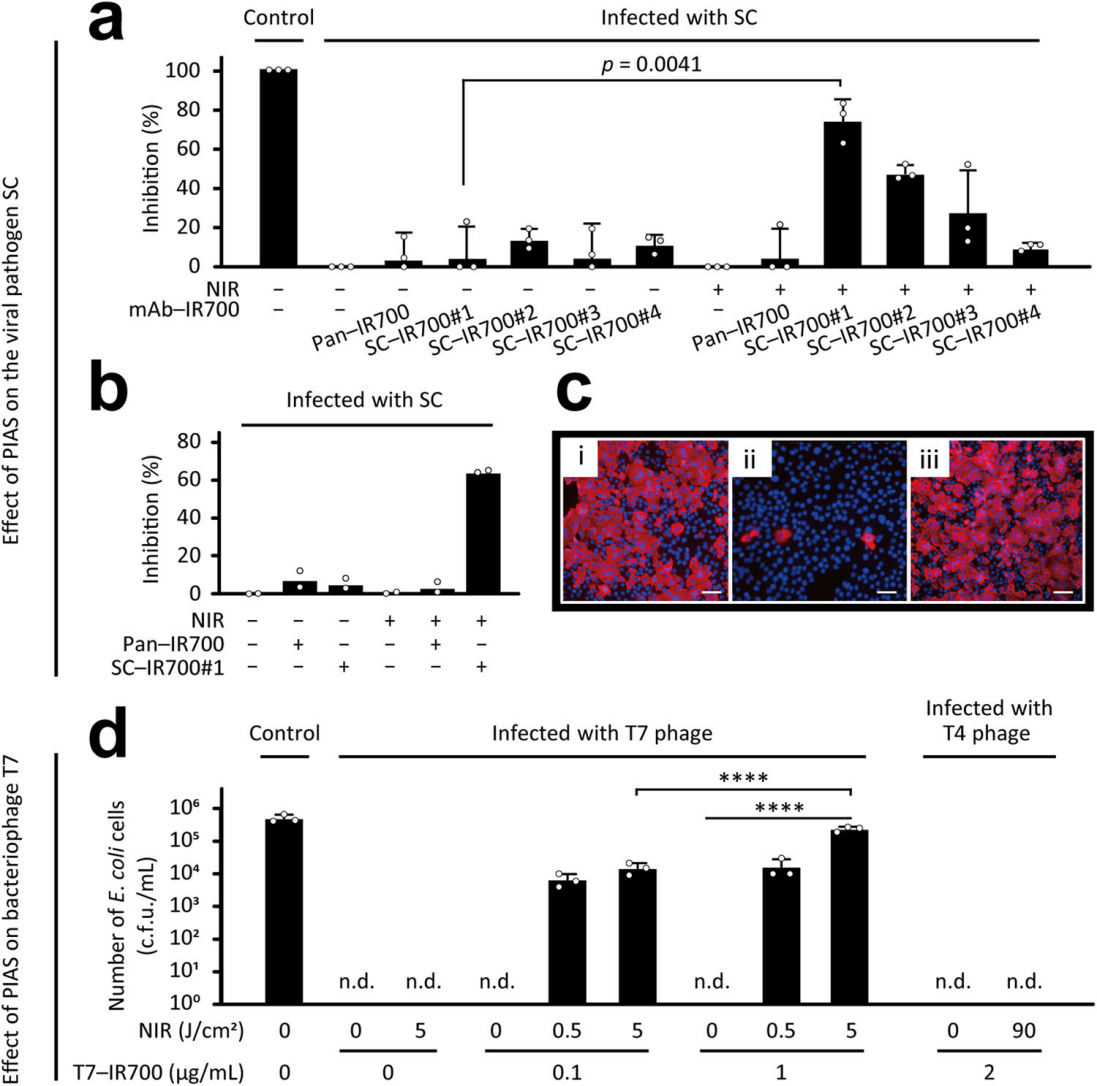
Virucidal effect of PIAS. **a**–**c** Virucidal effect of PIAS against SARS-CoV-2 (SC) was evaluated by assessing **a** the cell viability of VeroE6/TMPRSS2 cells and **b** viral RNA quantity after infection with PIAS-treated SC. Data normalised to the virus group are shown. SC-specific conjugates, SC–IR700#1, 2, 3, and 4; non-specific conjugates, Pan–IR700. Conjugates, 0.1 μg/mL; near-infra-red light (NIR) illumination, 10 J/cm^2^. **c** Immunostaining analysis of virus-infected cells in which an antibody against the SC-nucleocapsid protein was used (i–iii). SC treated with SC–IR700#1 without NIR (i); SC subjected to PIAS using SC–IR700#1 (ii); SC subjected to PIAS using Pan–IR700 (iii). SC and the nucleus are indicated in red and blue, respectively. The scale bar indicates 50 μm in (**c**). **d** Virucidal effect of PIAS using T7–IR700 conjugates against T7 phage was evaluated according to the cell viability of *Escherichia coli* cells using the colony counting method. Control, non-infected and untreated; c.f.u. colony-forming units, n.d. not detected. Mean and s.d. values are shown for triplicate samples; data were representative of at least three independent experiments. For statistical analyses, a two-tailed unpaired Student’s *t*-test (**a**, **b**) and ANOVA with Dunnett’s test (**d**) were performed. ****p < 0.0001.

To confirm a key property of PIAS, namely, the targeting of different microbes irrespective of species, we tested whether PIAS has a virucidal effect. We targeted the viral pathogen SC and tested the antiviral effect of PIAS using various anti-spike antibodies because viral spikes form oligomers on the viral envelope for glycosylation and intermolecular interactions (providing a variety of antigenicities). In addition, panitumumab (anti-EGFR)–IR700 conjugate (Pan–IR700) served as a non-targeting conjugate. PIAS using the SC-targeting conjugate SC–IR700#1 potently inactivated the pathogen, resulting in cell survival similar to that of non-infected cells (Fig. 4a). PIAS using SC–IR700#2 or 3 also inactivated SC (Fig. 4a); however, PIAS using SC–IR700#4 showed no significant effect on the pathogen (Fig. 4a), likely because of the characteristics of the antibody, such as specificity. Notably, PIAS showed an antiviral effect against SC, whereas the antibody conjugates had no neutralising activity against the pathogen in the test cases (Fig. 4a). No apparent effects were observed in cell samples treated with PIAS using the non-specific conjugate Pan–IR700 (Fig. 4a). The virucidal effect was also evaluated by quantifying the viral RNA (Fig. 4b), confirming that PIAS inactivated SC. In addition, SC-infected cells were subjected to immunostaining analysis^17^, indicating that almost all cells were infected with SC in (i) the untreated group and (ii) the group treated with PIAS using the non-specific conjugate Pan–IR700, but not (iii) those in the group treated with PIAS using the SC-targeting conjugate SC–IR700 (Fig. 4c).

Moreover, the bacterial virus bacteriophage T7^18^ was inactivated by PIAS using a T7-targeting conjugate (Fig. 4d), confirming that PIAS targets various microbes, regardless of their species.

### In vivo effects of PIAS

**Fig. 5:**
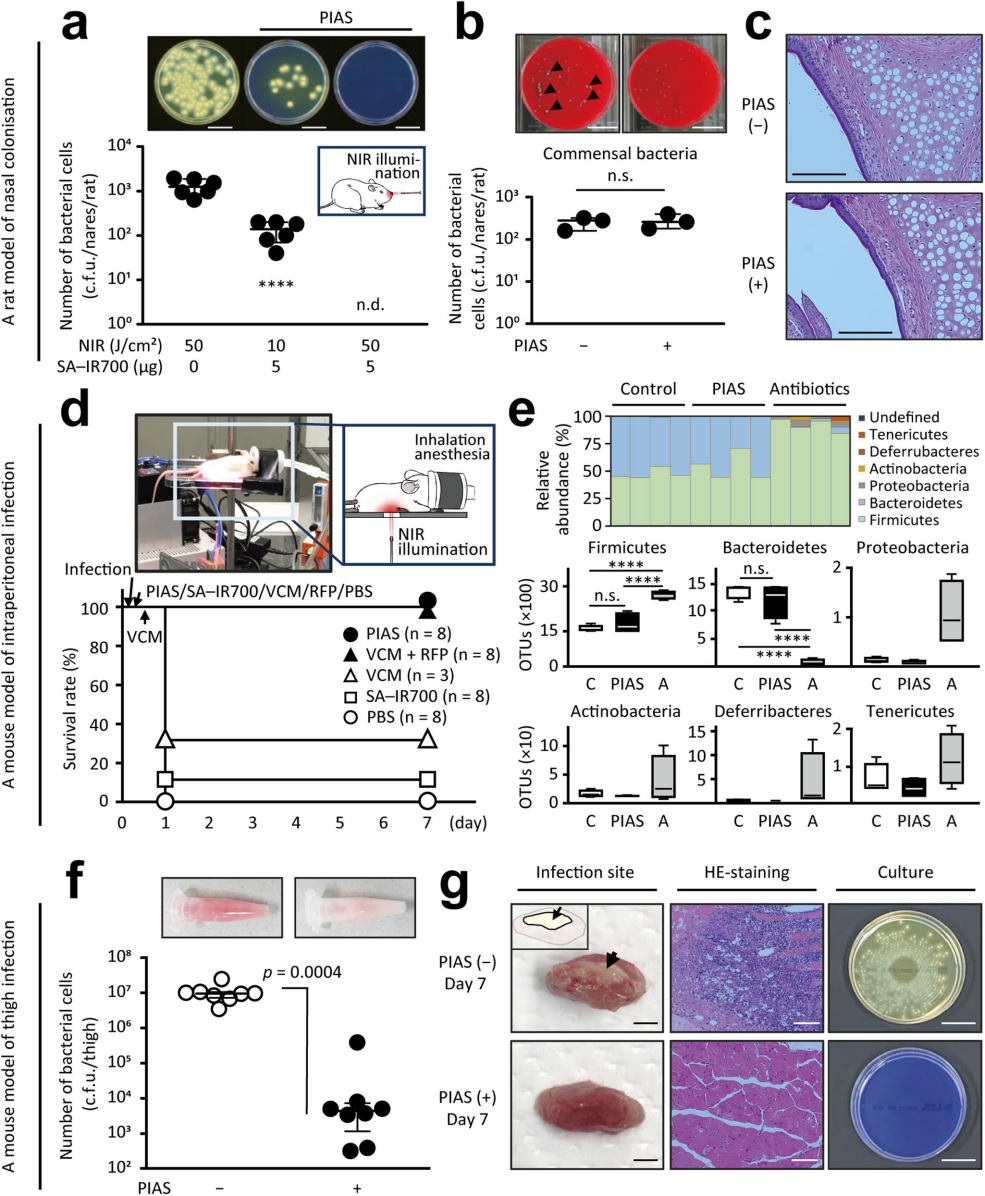
In vivo effects of PIAS. **a**–**c** Effect of PIAS on **a** methicillin-resistant *Staphylococcus aureus* (MRSA), **b** non-target commensal bacteria, and **c** nasal tissues of cotton rats colonised by MRSA. The illustrated image of PIAS against SA nasal colonisation is shown in **a**. NIR laser light was delivered from outside of the nares of cotton rats using laser fibres. **b** Arrowheads indicate MRSA colonies with haemolytic plaques. **c** Histological analysis of untreated and PIAS-treated nasal tissues. Scale bars indicate 3 cm (**a**, **b**) and 100 µm (**c**), respectively. **d**, **e** Effects of PIAS and antibiotics on a mouse MRSA intraperitoneal infection (survival analysis, **d**) and on normal host intestinal microflora (16 S rRNA-targeting metagenome analysis of bacterial phyla, **e**). A mouse undergoing PIAS treatment is shown. Control (C), phosphate-buffered saline; antibiotics (A), vancomycin (VCM) + rifampicin (RFP). Box elements: centre lines, medians; box limits, upper and lower quartiles; whiskers, minimum and maximum values. **f**, **g** Effect of PIAS on a mouse MRSA-thigh infection. **f** Homogenised thigh samples (day 1; top images) and colony counts. **g** On day 7, visual analysis (left), histochemical analysis (middle), and bacterial culture (right) were performed on the thigh samples. Arrows and the illustrated image (top left) indicate abscesses. PIAS conditions: SA–IR700 conjugate, 5 µg/animal; near-infra-red light (NIR) illumination, 50 J/cm^2^. SA–IR700 was used in both PIAS (−) and (+) groups. Scale bars indicate 2 mm (left), 100 µm (middle), and 3 cm (right) in (**g**), respectively. HE-staining haematoxylin-eosin staining, c.f.u. colony-forming units, n.d. not detected, n.s. not significant (*p* > 0.05). Median and IQR values are shown. Statistical analyses included ANOVA with the Sidak test (**a**), one-way ANOVA with the two-stage step-up method of Benjamini, Krieger, and Yekutieli (**e**), and a two-tailed unpaired Student’s *t*-test (**f**). ****p < 0.0001.

Finally, to confirm whether PIAS can specifically act on a target pathogen without affecting the normal host microflora in vivo, we used a rat model of MRSA-nasal colonisation^19^. Consistent with the in vitro results, PIAS eradicated the pathogen (Fig. 5a) without affecting commensal bacteria (Fig. 5b and Supplementary Fig. 14) and the nasal tissue (Fig. 5c and Supplementary Fig. 15) of rats, whereas NIR or conjugate treatment alone did not (Fig. 5a and Supplementary Fig. 16). Further analysis using a mouse model of MRSA intraperitoneal infection^20^ showed that PIAS eliminated the pathogen (Fig. 5d, e). No significant differences were observed in the normal intestinal microflora with PIAS treatment (Fig. 5d, e and Supplementary Fig. 17), suggesting that the treatment effect of PIAS does not cause dysbiosis^21^.

Additionally, PIAS was found to eliminate MRSA in the deep tissues of mice with MRSA-thigh infections^22^ (Fig. 5f, g). However, in untreated mice, hyperaemia was observed in homogenised thigh samples on day 1 (top images, Fig. 5f), whereas apparent abscesses (left images), inflammatory cell infiltration (middle images, and Supplementary Fig. 18), and persistent MRSA colonies (right images) were observed in day-7 thigh samples (Fig. 5g).

## Discussion

This study demonstrated that PIAS can be used as an antimicrobial tool based on both its target precision and flexibility against a broad range of microbial pathogens regardless of their species or drug-resistance status. PIAS enables the use of multiple conjugates against different epitopes, which can cover epitope variations and multiple target pathogens. Notably, the observed antimicrobial effects of PIAS were achieved even when using a commercial mAb that exhibited no neutralising activity. PIAS showed the target elimination of different microbes, including bacterial, fungal, and viral pathogens; other microbes, such as protozoan parasites without available drugs^23^, may also be targeted using PIAS.

For PIAS to be effective in vivo, conjugates and NIR must reach the site of infection; however, the treatment can be limited to complex tissues and biofilms. Conjugates should be administered into the target infection by intravenous or local injection. External NIR illumination can reach the target infection in complex tissues of patients with head-neck cancer, which was confirmed by our original photoimmunotherapy in clinical trials [ClinicalTrials.gov Identifier: NCT02422979 (phase I/II) and NCT03769506 (phase III)], where it was approved as a novel anticancer therapy in Japan in 2020. Endoscopy with a NIR laser can also be used for internal illumination, including in the lumen of the gastrointestinal tract and in deeper tissues.

Since PIAS requires antibodies against target pathogens, the preparation of a library of conjugates against some of the main pathogens can be useful for future situations. PIAS may be applied to future emerging infections if antibodies are available; however, the usefulness of conventional antimicrobial drugs is evident, at least in the current situation^1^. With regards to the aspect of clinical application, PIAS can be applied to patients who have already been treated with antimicrobials in clinics or with conventional approaches in hospitals and in whom treatment has failed, rather than as the first-line treatment. Considering that no panacea exists for antimicrobial strategies, choices must be made depending on the specific situation.

In conclusion, we demonstrated that PIAS showed targeted elimination of different microbial pathogens, irrespective of their species or drug-resistance status. PIAS can be used as a biological tool for selectively eliminating targeted/unwanted microbial cells or cells expressing different molecules in cell populations^24^, which may facilitate studies on the functions of each molecule, as well as the development of next-generation antimicrobial therapies.

## Methods

### Microbes and culture conditions

Various strains of *Staphylococcus aureus* (SA), including methicillin-sensitive SA (MSSA, JKmsSA1 and JCM2874), methicillin-resistant SA (MRSA, JKmrSA1, N315 and USA300), mupirocin-resistant MRSA (JKmmrSA1 resistant to mupirocin 62.5 µg/mL), and VISA (MU3 and MU50), were used. *Staphylococcus epidermidis* (JCM2414 and JKse1), *Staphylococcus capitis* JCM2420, *Staphylococcus microti* (an isolate from the nares of a cotton rat), *Corynebacterium tuberculostearicum* JKCt1 (an isolate from the nares of a healthy volunteer), *Candida albicans* (CA) [JCM1452 and JCM2085 with resistance to azole drugs (fluconazole, itraconazole, and voriconazole), JKfarCA1 with resistance to azole drugs and 32 µg/mL flucytosine], *Candida stellata* NBRC0703, *Saccharomyces cerevisiae* DYSc, and *Escherichia coli* DH5α were also used. Furthermore, T7 (NBRC20007) and T4 (NBRC20004) phages were used. Trypticase soy broth (TSB), brain–heart infusion broth, L broth, mannitol salt agar with egg yolk, TSB agar supplemented with rabbit blood cells, and OPAII *Staphylococcus* agar were obtained from BD Biosciences (Franklin Lakes, NJ, USA). TSB containing 0.5% glucose, Sabouraud broth, and equine defibrinated blood (Kohjin Bio, Saitama, Japan) was used. CROMagar was obtained from Kanto Chemical Co. Ltd. (Tokyo, Japan). In addition to cells in the exponential phase, microbial cells in the stationary phase are used; the stationary phase induces the development of persister cells that are recalcitrant to antibiotics^25^. Bacterial and fungal cells were cultured at 37 °C with shaking for 16 h (stationary phase). To obtain microbial cells in the exponential phase, the cells were harvested at an OD of 0.4, which was determined with a photometer (Mini Photo 518 R; TAITEC, Saitama, Japan).

VeroE6/TMPRSS2 cells (JCRB #1819)^17^ were cultured in DMEM containing 10% foetal bovine serum. SARS-CoV-2 (SC; JPN/TY/WK-521/2020, EPI_ISL_408667)^17^ was obtained from National Institute for Infectious Disease (Tokyo, JAPAN), and handled in a biosafety level 3 laboratory. All experiments with a clinical isolate of SC were performed within a biological safety cabinet with prior approval from the biosafety committee of Yokohama City University.

### Reagents

The anti-SA monoclonal antibody (mAb) against the SA peptidoglycan epitope (clone Staph12-569.3, murine IgG3) was purchased from QED Bioscience (San Diego, CA, USA). Anti-CA mAb (clone MC3, murine IgG3), which recognises the putative β-1,2-mannan epitope in the cell wall mannoproteins and phospholipomannans of CA, was purchased from ISCA Diagnostics (Exeter, UK). The anti-SC spike mAb was obtained from GeneTex (GTX632604; Irvine, CA, USA). Anti-T7 phage mAb (T7·Tag antibody, murine IgG2b) directed against the 11 amino acid gene 10 leader peptide (MetAlaSerMetThrGlyGlyGlnGlnMetGly) of T7 phage was purchased from Merck KGaA (Darmstadt, Germany). Anti-human epidermal growth factor receptor 2 (HER2) mAb trastuzumab (Herceptin, humanised IgG1) was purchased from Chugai Pharmaceutical (Tokyo, Japan). IRDye700DX (IR700) was purchased from LI-COR Biosciences (Lincoln NE, USA). RPMI 1640 medium without phenol red was purchased from Thermo Fisher Scientific (Waltham, MA, USA).

### Synthesis and purification of IR700-conjugated mAb

IR700-conjugating mAb was synthesised as previously described^11^. Briefly, the mAb (1.0 mg, 6.8 nmol) was incubated with IR700 (66.8 µg, 34.2 nmol) in 0.1 M Na_2_HPO_4_ (pH 8.5) at 25 °C for 1 h. The mixture was purified on a Sephadex G50 column (PD-10; GE Healthcare, Little Chalfont, UK). To confirm the number of fluorophore molecules conjugated to each mAb molecule, the concentrations of protein and IR700 were measured spectroscopically based on their absorption at 280 and 689 nm, respectively (UV-1800; Shimadzu, Kyoto, Japan). Conjugates containing approximately three IR700 molecules per mAb molecule were used.

### Binding of mAb–IR700 conjugates to microbial cells

mAb–IR700 conjugate (1 µg) was added to 100 µL of a microbial suspension containing ~1 × 10^5^ colony-forming units (c.f.u.) and incubated for 1 h at 4 °C, followed by washing the cells with 3 mL of RPMI twice. The fluorescence of IR700 was measured with a flow cytometry analyser (MACSQant analyser; Miltenyi Biotec, Bergisch Gladbach, Germany) and fluorescence microscopy (IX73; Olympus, Tokyo, Japan) with the following filter settings: 608–648-nm excitation filter and 672–712-nm emission filter. To confirm the target specificity of mAb–IR700 conjugate, unconjugated mAb was added before mAb–IR700 treatments.

### SEM analyses

Scanning electron microscopy (SEM) analyses were performed to detect mAb binding to the bacterial cells. The SA–IR700 conjugate-treated SA JKmrSA1 cell suspension was washed with and resuspended in phosphate-buffered saline (PBS) containing 1% bovine serum albumin. Colloidal gold (12 nm) goat anti-mouse IgG (Jackson ImmunoResearch Laboratories, West Grove, PA, USA) (1: 25) was added to the solution and incubated for 1 h at room temperature (25 °C). The mixture was dropped onto a nano-percolator to remove unbound antibodies and then washed with PBS. The samples were analysed using SEM (SU8010; Hitachi, Tokyo, Japan). PIAS-treated cells were subjected to SEM analysis.

### mAb–IR700 conjugates mediate PIAS activity in vitro

mAb–IR700 conjugates (0.01 to 20 µg) were added to ~1 × 10^5^ c.f.u. of microbial suspension (100 µL of total volume) and incubated for 1 h at 4 °C in the dark. Microbial cells were then irradiated with near-infra-red (NIR) illumination (5–90 J/cm^2^) using a light-emitting diode releasing light at 670–710 nm (L690-66-60; Epitex, Kyoto, Japan)^11,26,27^. A power density of 24 mW/cm^2^ was measured with an optical power metre (PM 100, Thorlabs, Newton, NJ, USA). Serially diluted samples were plated on agar plates for overnight culture to determine microbial viability.

### PIAS for SC

SC (multiplicity of infection = 0.05) was treated with anti-SC spike-IR700 conjugate (0.1 µg/mL) at 37 °C for 10 min in the dark and then divided into two samples. One sample was treated with NIR illumination (10 J/cm^2^), and duplicates were left untreated. For the infection assay, VeroE6/TMPRSS2 cells seeded into 96-well plates were infected with NIR-treated or untreated viruses. The cells were washed to remove the unbound virus at 2 h post-infection. Two days after infection, the supernatant was collected and RNA was extracted using a QIAamp viral RNA Mini Kit (Qiagen, Hilden, Germany). Cell viability was measured in the form of ATP present in the culture supernatants after virus lysis. The viral RNA was quantified using a CFX96 Real-Time System (Bio-Rad Laboratories, Hercules, CA, USA) with TaqMan Fast Virus 1-Step Master Mix (Thermo Fisher Scientific) using 5′-AAATTTTGGGGACCAGGAAC-3′ and 5′-TGGCAGCTGTGTAGGTCAA-3′ as the primer set and 5′-FAM-ATGTCGCGCATTGGCATGGA-BHQ-3′ as the probe^28^. Infected cells were washed and 40 µL of CellTiter-Glo Substrate (Promega, Madison, WI, USA) was added and cell viability was measured with the GloMax Discover System (Promega). The percentage inhibition was calculated using the formula: (RLU − virus-infected cells RLU)/(uninfected cells RLU − infected cells RLU) × 100.

In addition, the antiviral effect of PIAS on SCs was evaluated by immunostaining. The cells were fixed with 4% paraformaldehyde, permeabilised with 0.5% Triton X-100, and blocked with Blocking One (Nacalai, Tokyo, Japan) at room temperature for 15 min. The cells were incubated with a polyclonal antibody against SC-nucleocapsid protein (NB100-56576; Novus Biologicals, Littleton, CO, USA) (1: 100) at room temperature for 1 h. After incubation, the cells were stained with Alexa 568-labelled anti-rabbit antibody (1: 1000, Thermo Fisher Scientific) for 1 h at room temperature. The nuclei were stained with ProLong Gold Antifade Mountant with DAPI (Thermo Fisher Scientific). Images were captured and measured using an imager (BZ-9000; Keyence, Tokyo, Japan).

### PIAS for bacterial viruses

T7 and T4 phages (10^9^ plaque-forming units (p.f.u.)/test) were treated with T7–IR700 conjugate (0.1–2 µg/test) at 4 °C for 1 h and then divided into two samples. One sample was treated with NIR illumination, whereas the other was left untreated. Both samples were co-cultured with *E*. *coli* DH5α or BL21 cells at 37 °C for 20 min. After co-culture, the samples were cultured on agar plates, and bacterial colonies were enumerated.

Notably, when phages (10^6^ p.f.u./test) were co-cultured with *E*. *coli* cells (10^6^ c.f.u./test), colonies were observed. In contrast, no colonies were observed when phages (10^9^ p.f.u./test) were used alone. As an antiviral effect was clearly observed, we adopted this method in our study.

### In vivo studies

Animal studies were performed in accordance with the guidelines established by the Animal Care Committee of the Jikei University School of Medicine. All in vivo experiments were performed under isoflurane anaesthesia.

### PIAS in the in vivo colonisation model

The cotton rat nasal colonisation model^19^ was used to determine the feasibility of the PIAS in vivo. Six- to ten-week-old cotton rats (*Sigmodon hispidus*) were obtained from the Animal Research Center of the University of Occupational and Environmental Health School of Medicine (Fukuoka, Japan). All rats were allowed to acclimatise and recover from shipping-related stress for one week and kept under a controlled light/dark cycle (12 h: 12 h) before the experiments. Ten microlitres of a suspension of 1 × 10^6^ c.f.u. MRSA JKmrSA1 cells were instilled in both cotton rat nares. The animals were then kept in a supine position for approximately 1 h to recover from anaesthesia. At 7 days after intranasal instillation, the animals were randomised into five groups, with at least three rats per group, which were treated as follows: (i) intranasal administration of PBS (10 µL); (ii) NIR laser illumination (50 J/cm^2^); (iii) intranasal administration of PBS (10 µL), followed by NIR laser illumination (50 J/cm^2^); (iv) intranasal administration of SA–IR700 conjugates (5 µg, 10 µL), followed by NIR laser illumination (10 J/cm^2^); or (v) intranasal administration of SA–IR700 conjugates (5 µg, 10 µL), followed by NIR laser illumination (50 J/cm^2^).

All procedures were performed under anaesthesia, and NIR laser illumination was performed at 1 h after intranasal administration of SA–IR700 or PBS. NIR laser illumination was performed with a 690-nm continuous-wave laser at a power density of 330 mW/cm^2^ (ML6540-690; Modulight, Tampere, Finland). After NIR laser treatment, the animals were sacrificed, and the face, specifically the exterior of the nose, was carefully disinfected with 70% alcohol. Anterior nares were harvested by dissecting the nose. Harvested nasal samples were collected in 1.5 mL of PBS containing 10 µL of Tween 20 and vortexed at the maximum speed for 1 min. Serially diluted samples were plated on agar plates for overnight culture to determine bacterial viability. The absence of SA contamination was confirmed in all animals before use by nasal and rectal swab cultures.

### PIAS in an intraperitoneal infection model

A mouse model of intraperitoneal infection^20^ was used. Five- to seven-week-old female-specific pathogen-free BALB/c mice were obtained from CLEA Japan (Tokyo, Japan). The mice were intraperitoneally injected with 100 µL suspension of 2 × 10^8^ c.f.u. MRSA JKmrSA1 cells and treated with PIAS or antibiotics [vancomycin (VCM), rifampicin (RFP)]. The following groups were used: (i) PIAS-treated group (*n* = 8): SA–IR700 (50 µg/mouse) and NIR illumination of the entire abdomen (50 J/cm^2^) from outside the body; (ii) PIAS control group (*n* = 8): SA–IR700 (50 µg/mouse) without NIR illumination; (iii) VCM-treated group (*n* = 3): VCM 10 mg/kg; (iv) RFP-and VCM-treated group (*n* = 8): RFP at 100 mg/kg and VCM at 50 mg/kg; and (v) control (*n* = 3): 100 µL PBS. SA–IR700 and VCM or RFP were administered intraperitoneally or orally, respectively. Treatments were performed within 1 h after infection once during the experimental period, whereas only VCM was administered twice. Survival and adverse events were monitored via a once-daily assessment for 7 days. Faeces of PIAS and antibiotic (VCM and RFP)-treated mice and those of the non-treated mice were used for 16S-targeting metagenome analysis.

### PIAS in the murine thigh infection model

A mouse model of thigh infection^22^ was used. A 100-µL suspension of 1 × 10^7^ c.f.u. of MRSA JKmrSA1 cells was injected into the right thigh muscle of six- to seven-week-old female mice, which were then treated with or without PIAS (*n* = 8 per group). The PIAS conditions were as follows: SA–IR700 (50 µg/mouse) and NIR illumination (50 J/cm^2^) of the right thigh from outside the body. The control mice were treated with SA–IR700 (50 µg) without NIR illumination. One day after treatment, the mice were sacrificed with cervical dislocation, and the right thighs were dissected. Thigh samples were collected in PBS containing Tween 20 and homogenised manually. The homogenised samples were cultured on OPAII *Staphylococcus* agar to evaluate the bactericidal effect of PIAS on the pathogen cells in the thigh. Thigh samples obtained 7 days after treatment were used to assess the pathology. Macroscopic findings were confirmed histologically, as appropriate, with haematoxylin-eosin staining.

### Metagenomic analysis

DNA was extracted from faecal pellets, and PCR was performed using 27Fmod 5′-AGRGTTTGATYMTGGCTCAG-3′ and 338 R 5′-TGCTGCCTCCCGTAGGAGT-3′ to the V1–V2 region of the 16 S rRNA gene^29,30^. 16 S metagenomic sequencing was performed using MiSeq according to the Illumina protocol (San Diego, CA, USA). Two paired-end reads were merged using the fastq-join programme based on overlapping sequences. Reads with an average quality value <25 and inexact matches to both universal primers were filtered out. Filter-passed reads were further analysed after trimming off both the primer sequences. For each sample, 3000 quality filter-passed reads were rearranged in descending order according to the quality value and then clustered into operational taxonomic units with a 97% pairwise identity cut-off using the UCLUST programme (Edgar 2010) version 5.2.32 (https://www.drive5.com). Taxonomic assignment of each operational taxonomic unit was performed by searching for similarities against the RDP and NCBI genome databases using the GLSEARCH programme.

### Statistical analyses

Mean ± standard deviation (s.d.) values were calculated from a minimum of three samples. Calculations and statistical analyses were performed using GraphPad Prism software (ver. 8; GraphPad, Inc., La Jolla, CA, USA) and Excel software (Microsoft, Redmond, WA, USA). A two-sided Student’s *t*-test was used to determine the differences in bacterial counts between the two treatment groups. One-way analysis of variance (ANOVA) with Dunnett’s test or the Sidak test or two-way ANOVA with the Sidak test was performed for multiple group comparisons. The Kaplan–Meier survival curve was assessed using the log-rank (Mantel-Cox) test. In metagenome analysis, ANOVA was performed for multiple group comparisons, and any significant differences were evaluated using the two-stage step-up method of Benjamini, Krieger, and Yekutieli^31^. Results were considered statistically significant at a *p* value < 0.05.

### Reporting summary

Further information on research design is available in the Nature Research Reporting Summary linked to this article.

### Supplementary information

Supplementary Information

Description of Additional Supplementary Files

Supplementary Data 1

Reporting Summary

## Data Availability

Data of this study are available from the corresponding authors upon reasonable request. Source data are available in Supplementary Data 1 or 10.6084/m9.figshare.19776094. The raw 16S metagenomic data have been deposited to DDBJ Sequence Read Archive accession number DRA014325 in the DDBJ BioProject database.
