# Supplementary material for: Antimicrobial strategy for targeted elimination of different microbes, including bacterial, fungal and viral pathogens
Source: Commun Biol. 2022 Jul 4;5:647. doi: 10.1038/s42003-022-03586-4 (PMC9253063; doi:10.1038/s42003-022-03586-4)
Supplement: Supplementary file 1 — Supplementary Information [file 42003_2022_3586_MOESM1_ESM.pdf]

Supplementary Information for

Antimicrobial strategy for targeted elimination of different microbes, including bacterial, fungal and viral pathogens

Makoto Mitsunaga[1*†], Kimihiro Ito[1*], Takashi Nishimura[1], Hironori Miyata[2], Kei Miyakawa[3], Takeshi Morita[3], Akihide Ryo[3], Hisataka Kobayashi[4], Yoshimitsu Mizunoe[5], Tadayuki Iwase[6†]

[1] Division of Gastroenterology and Hepatology, Department of Internal Medicine, The Jikei University School of Medicine, Tokyo, Japan.
[2] Animal Research Center, School of Medicine, University of Occupational and Environmental Health, Kitakyushu, Japan.
[3] Department of Microbiology, Yokohama City University School of Medicine, Kanagawa, Japan.
[4] Molecular Imaging Program, Center for Cancer Research, National Cancer Institute, NIH, Bethesda, USA.
[5] The Jikei University School of Medicine, Tokyo, Japan.
[6] Research Center for Medical Sciences, The Jikei University School of Medicine, Tokyo, Japan
[*] These authors contributed equally.
[†] Corresponding authors.

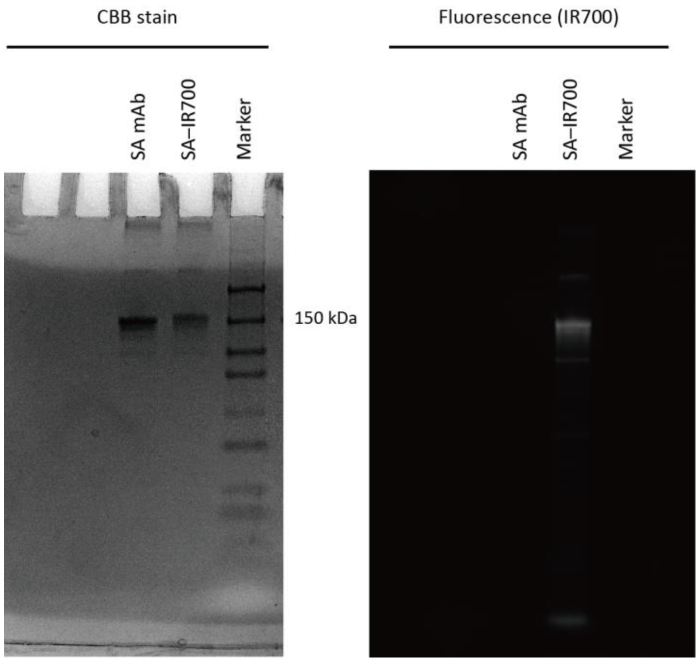

Supplementary Figure 1. IR700 fluorescence derived from mAb–IR700 conjugates. SA–IR700 conjugates and anti-*Staphylococcus aureus* monoclonal antibodies (SA mAbs) were subjected to non-reducing SDS-PAGE. The gel was stained with Coomassie Brilliant Blue (CBB) to obtain protein signal with ChemiDoc Touch (BioRad, left image). The same gel was observed with IVIS imaging system (Perkin Elmer) to obtain IR700 fluorescence (right image), corresponding to the band of SA–IR700 conjugates in the gel. Note that these are the raw/uncropped images and the size marker was not detected with fluoresce imaging. Images represent at least three independent experiments.

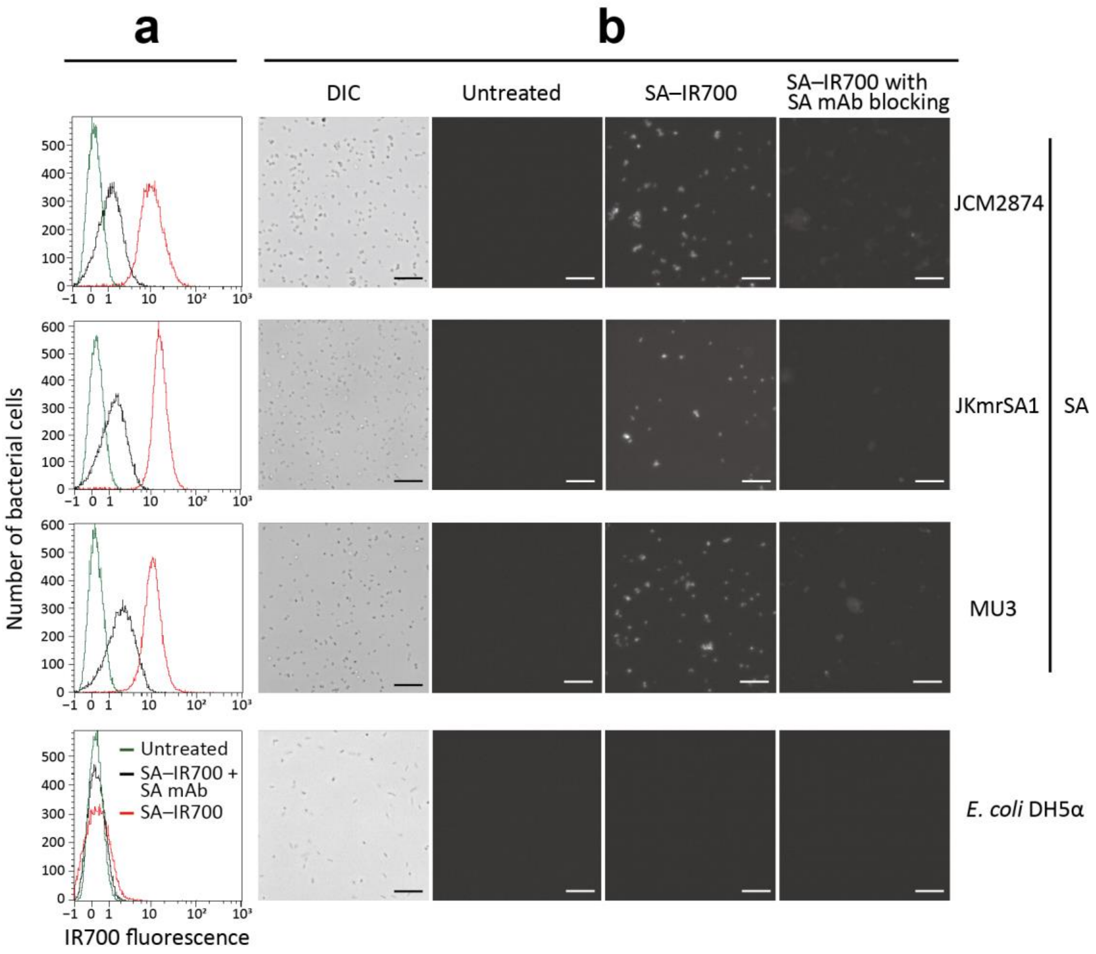

Supplementary Figure 2. Binding of mAb-conjugates to the target cells. The binding ability of SA–IR700 conjugates with *Staphylococcus aureus* (SA; JCM2874, JKmrSA1, and MU3) and *Escherichia coli* (DH5α) cells was examined using (**a**) flow cytometry and (**b**) fluorescence microscopy. SA–IR700 conjugates (2 µg/test) and SA mAb (20 µg/test) were used. **a** Various SA strains treated with SA–IR700 conjugates or SA–IR700 conjugates with SA mAb blocking were subjected to flow cytometric analysis. The y-axis indicates the number of bacterial cells and x-axis indicates the fluorescence of IR700. **b** Bacterial cells treated with SA–IR700 conjugates or SA–IR700 conjugates with SA mAb blocking were subjected to fluorescence or differential interference contrast (DIC) microscopic analyses. Bright spots indicate fluorescence derived from SA–IR700 conjugates. Scale bars indicate 20 µm. Data and images are representative of at least three independent experiments.

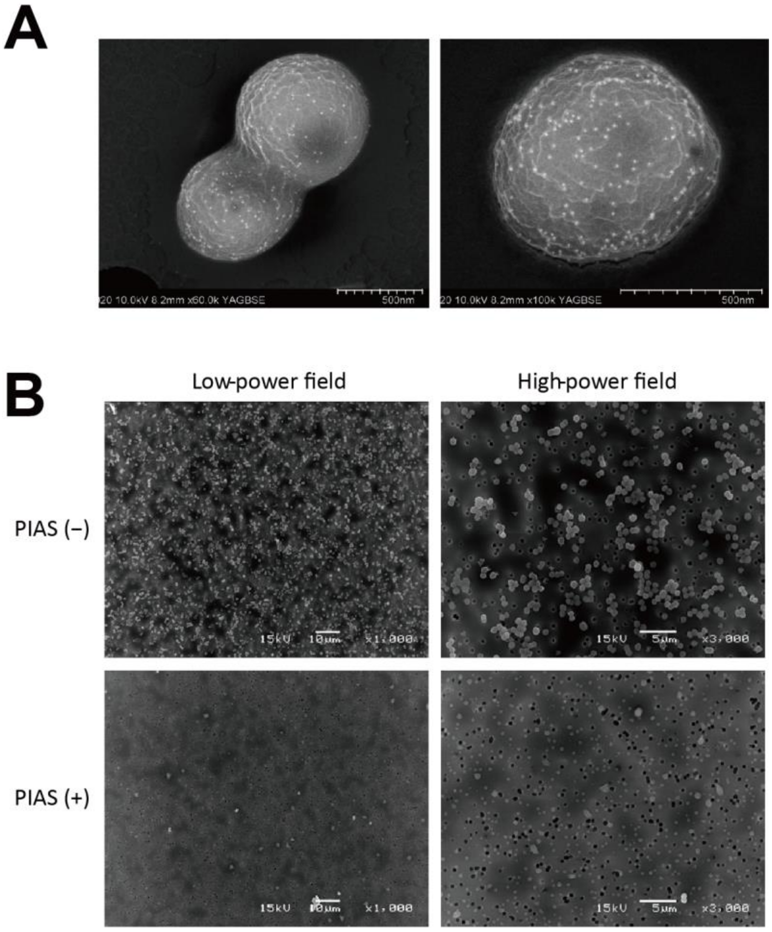

Supplementary Figure 3. Binding of conjugates to and disruption of targets. a *Staphylococcus aureus* (SA) JKmrSA1 cells were subjected to immunogold staining for SA–IR700 conjugate and then analysed using scanning electron microscopy (SEM). Conjugate- and colloidal gold secondary antibody-treated SA cells are shown. Bright dots on the SA cell surfaces indicate colloidal gold secondary antibodies binding to SA–IR700. **b** Injury to microbial cells by PIAS. SA cells subjected to near-infrared light (NIR) illumination (30 J/cm^2) were analysed using SEM. Upper images: PIAS (-), untreated control; lower images: PIAS (+), SA–IR700 with NIR-treated SA cells. Images represent at least three independent experiments.

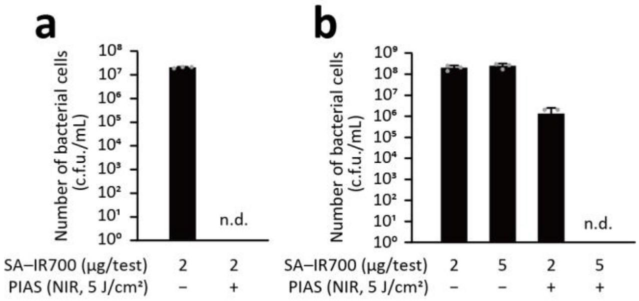

Supplementary Figure 4. Effects of PIAS on 10^7 and 10^8 c.f.u./mL of SA. **a**, **b** 10^7 (**a**) and 10^8 (**b**) c.f.u./mL of bacterial cells from *Staphylococcus aureus* (SA) JKmrSA1 were subjected to PIAS. c.f.u., colony-forming units; n.d., not detected. Data are representative of at least three independent experiments. Mean and s.d. values are shown for triplicate samples.

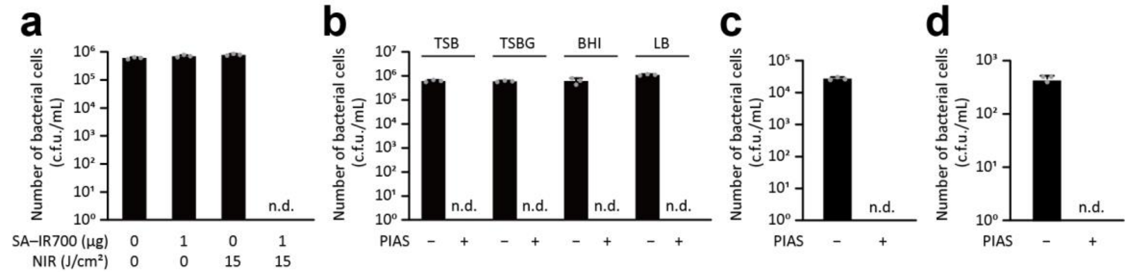

Supplementary Figure 5. Effect of PIAS on SA under various conditions. The bactericidal effect of PIAS on *Staphylococcus aureus* (SA) under various conditions was evaluated. **a** Bacterial cells harvested from the exponential phase were subjected to PIAS using SA–IR700 conjugate or treated with a sole conjugate or near-infrared light (NIR) illumination. **b** Bacterial cells cultured in various media were subjected to PIAS under the following conditions: SA–IR700, 1 µg/test; NIR illumination, 15 J/cm^2. TSB, tryptic soy broth; TSBG, tryptic soy broth supplemented with glucose (1%); BHI, brain-heart infusion broth; LB, L broth. **c** Bacterial cells were suspended in equine defibrinated blood and subjected to PIAS. The PIAS conditions were as follows: SA–IR700, 4 µg/test; NIR illumination, 30 J/cm^2. **d** The effect of PIAS on bacterial cells in 3T3 cells. Bacterial cells (1×10^7 cells/test) treated with SA–IR700 (30 µg/test) were co-cultured with 3T3 cells (confluent cells in 35-mm dish) and co-cultured at 37°C under 5% CO_2 for 30 min.[1,2] After co-culture, the cells were rinsed three times with 1 mL of RPMI, and extracellular SA was lysed with 1 mL of lysostaphin (FUJIFILM Wako Pure Chemical, Osaka, Japan) solution (1 mg/mL) for 30 min.[1] The cells were rinsed five times with 1 mL of RPMI and subjected to NIR illumination. After treatment, bacterial cells were homogenised and cultured on agar plates. The PIAS conditions were as follows: SA–IR700, 4 µg/test; NIR illumination, 30 J/cm^2. (**b**–**d**) SA–IR700 was used in both PIAS (−) and (+) groups. c.f.u., colony-forming units; n.d., not detected. Data are representative of at least three independent experiments. Mean and s.d. values are shown for triplicate samples.

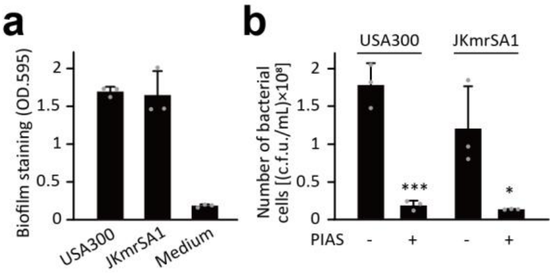

Supplementary Figure 6. Effects of PIAS on SA biofilms. Bacterial cells of *Staphylococcus aureus* (SA) JKmrSA1 and USA300 were used. To induce biofilm formation, SA cells were statically cultured in TSBG in the wells of a 96-well microtitre plate at 37°C for 8 h. To remove the deposited SA cells (non-biofilm cells), SA biofilms were washed five times with RPMI and then were subjected to the following tests. **a** SA biofilms were quantified using the conventional crystal violet staining method[3,4] to investigate whether our samples were biofilms. Medium (without bacterial cells) was used as a negative control. **b** Effect of PIAS on SA cells in their biofilms. After PIAS, the SA biofilms in the wells were scraped using a small scraper and were homogenised. The samples were cultured on agar plates at 37°C for 16 h. The bactericidal effect was evaluated using the colony-counting method. The PIAS conditions were as follows: SA–IR700, 10 µg/test; NIR illumination, 50 J/cm^2. c.f.u., colony-forming units.

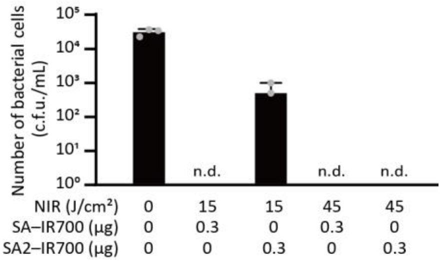

Supplementary Figure 7. Bactericidal effects of PIAS with an anti-SA antibody against different epitopes. *Staphylococcus aureus* (SA) JKmsSA1 cells were subjected to PIAS using an anti -SA monoclonal antibody [clone Staph12-569.3 (SA) or clone Staph11-232.3 (SA2)]–IR700 conjugate; SA2 was only used in this experiment. After PIAS, the bacterial cells were cultured on agar plates and colonies were enumerated to evaluate the bactericidal effect. NIR, near-infrared light; c.f.u., colony-forming units; n.d., not detected. Data are representative of at least three independent experiments. Mean and s.d. values are shown for triplicate samples.

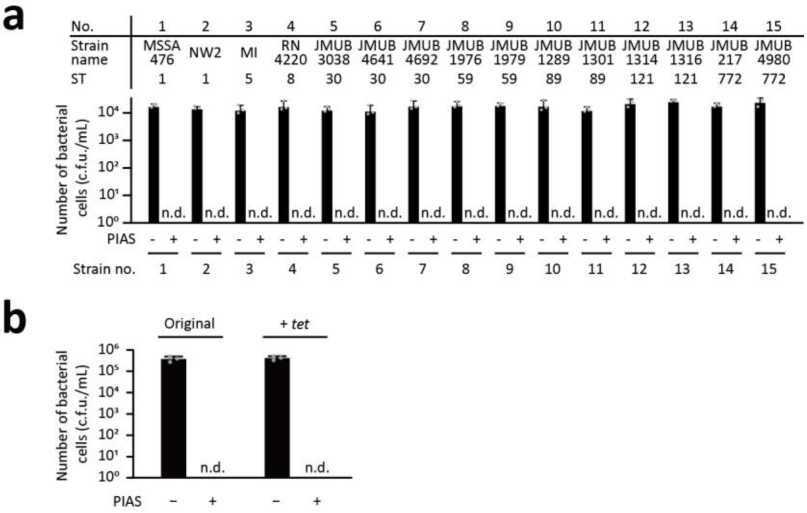

Supplementary Figure 8. Bactericidal effects of PIAS against various SA strains. a Diverse panel of *Staphylococcus aureus* (SA) sequence types was tested. Bacterial cells (approximately 10^5 c.f.u./test) were treated with the PIAS. **b** SA JKmsSA1 (original) cells and those of its derivative strain JKmsSA1 harbouring a plasmid encoding tetracycline-resistance gene (+ *tet*) were subjected to PIAS using the SA–IR700 conjugate. After PIAS, the bacterial cells were cultured on agar plates and colonies were enumerated to evaluate the bactericidal effect. SA–IR700 (2 µg/test) was used in both the PIAS (−) and (+) groups. c.f.u., colony-forming units; n.d., not detected. Mean and s.d. values are shown for triplicate samples.

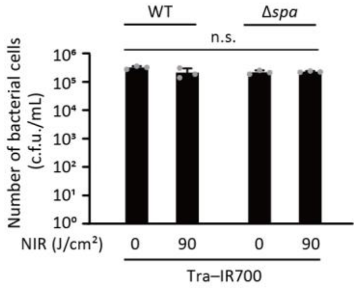

Supplementary Figure 9. Contribution of protein A to the bactericidal effect of PIAS on SA cells. *Staphylococcus aureus* (SA) cells with a deficiency in *spa* (Δ*spa*) and wild-type (WT, RN4220) were subjected to PIAS, and the bacterial samples were cultured on agar plates. *spa* encodes IgG-binding protein A. Bacterial viability was evaluated using the colony counting method. PIAS conditions: Tra–IR700 conjugate, 2 µg/test; near-infrared light (NIR) illumination, 90 J/cm^2. Tra–IR700 was used in both the PIAS (−) and (+) groups. c.f.u., colony-forming units; n.d., not detected; n.s., not significant ($p > 0.05$). Results were considered as statistically significant at a p value < 0.05. Data are representative of at least three independent experiments. Mean and s.d. values are shown for triplicate samples. Two-way ANOVA was performed.

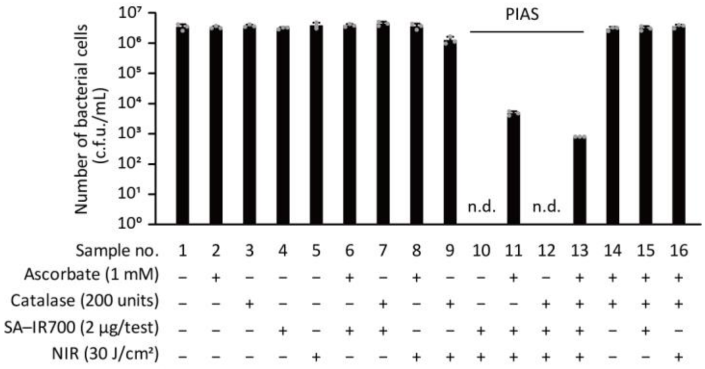

Supplementary Figure 10. Effect of ROS on bactericidal effect of PIAS. *Staphylococcus aureus* JKmsSA1 cells were subjected to PIAS in the presence of ascorbate (1 mM) and/or catalase (200 units/test); these agents showed antioxidant effects. After PIAS, bacterial cells were cultured on agar plates to evaluate their viability. NIR, near-infrared light. c.f.u., colony-forming units; n.d., not detected. Data are representative of at least three independent experiments. Mean and s.d. values are shown for triplicate samples.

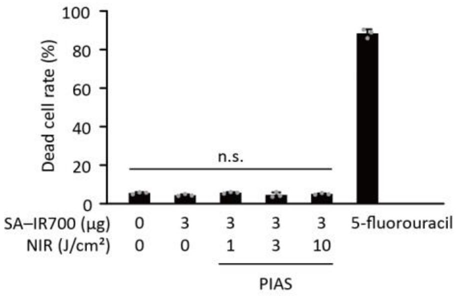

Supplementary Figure 11. Effect of PIAS on nontargeted fibroblast cells. The cytotoxic effect of PIAS using SA–IR700 conjugate on non-targeted fibroblast cells was investigated. Cells of the fibroblast cell line 3T3 were treated with SA–IR700 for 3 h and then exposed to near-infrared light (NIR) or 5-fluorouracil (5 µg/mL) for 24 h. 5-Fluorouracil is an anticancer drug exhibiting cytotoxicity. The cytotoxicity was evaluated using a live/dead assay. Columns indicate the mean values normalised to the control (untreated cells). n.s., not significant ($p > 0.05$).Data are representative of at least three independent experiments. The error bars represent the standard error of triplicate samples. One-way ANOVA was performed.

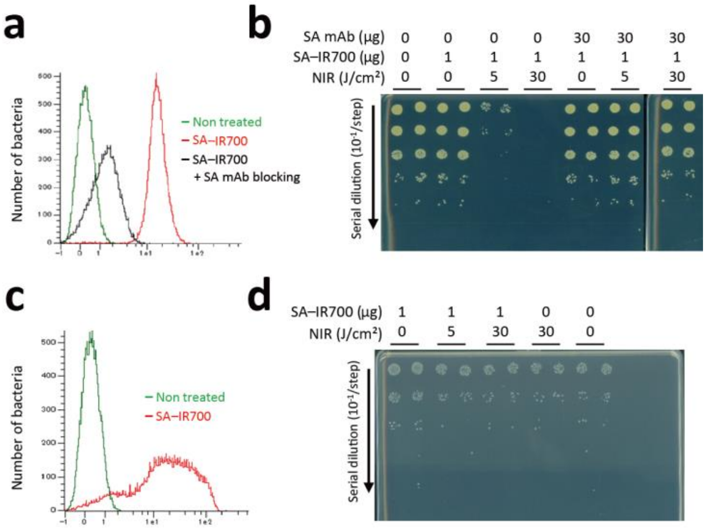

Supplementary Figure 12. Flow cytometric analysis of SA–IR700 conjugate-treated *Staphylococcus epidermidis* cells. **a**, **c** Binding ability of SA–IR700 conjugate toward (**a**) *Staphylococcus aureus* (SA) and (**c**) *Staphylococcus epidermidis* cells was examined using flow cytometry. The y-axis indicates the number of bacterial cells, and x-axis indicates the fluorescence of IR700. SA cells treated with SA–IR700 with SA mAb blocking are indicated by a black line (**a**). **b**, **d** SA (**b**), and *S. epidermidis* (**d**) cells subjected to PIAS using SA–IR700 were cultured to evaluate the bactericidal effect of PIAS. NIR, near-infrared light. Data and images are representative of at least three independent experiments.

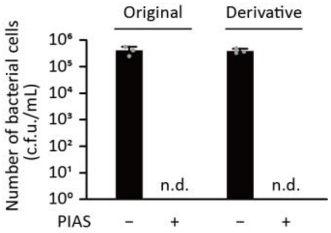

Supplementary Figure 13. Follow-up experiment to study the emergence of recalcitrant strains in PIAS. *Staphylococcus aureus* JKmsSA1 (original) and its derivative strain after 30 passages (derivative) were subjected to PIAS. Although no colonies were observed when PIAS was performed with bacterial cells at 10^6 c.f.u., some colonies were observed when PIAS was performed with bacterial cells at more than 10^8 c.f.u. Thus, PIAS eradicated the cells of such colonies/strains at 10^6 c.f.u. PIAS conditions: SA–IR700 conjugate, 2 µg/test; near-infrared light illumination, 90 J/cm^2. SA–IR700 was used in both the PIAS (−) and (+) groups. c.f.u., colony-forming units; n.d., not detected. Mean and s.d. values are shown for triplicate samples.

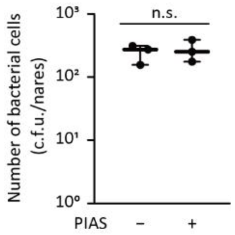

Supplementary Figure 14. Effect of PIAS on normal rat flora, including *Staphylococcus microti*. Animals colonised by MRSA JKmrSA1 were subjected to PIAS using the SA–IR700 conjugate. Subsequently, the samples were cultured on mannitol salt agar containing egg yolks. PIAS conditions: SA–IR700 conjugate, 5 µg/animal; near-infrared light illumination, 50 J/cm^2. SA–IR700 was used in both the PIAS (−) and (+) groups. c.f.u., colony-forming units; n.s., not significant (p > 0.05). Median and IQR values are shown. A two-tailed unpaired Student's t-test. A two-tailed unpaired Student's *t*-test was performed.

a

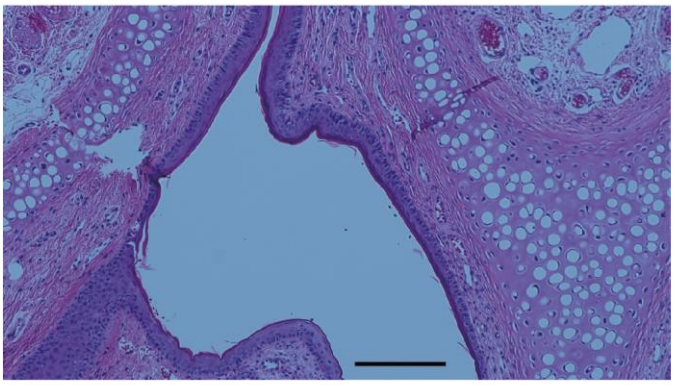

b

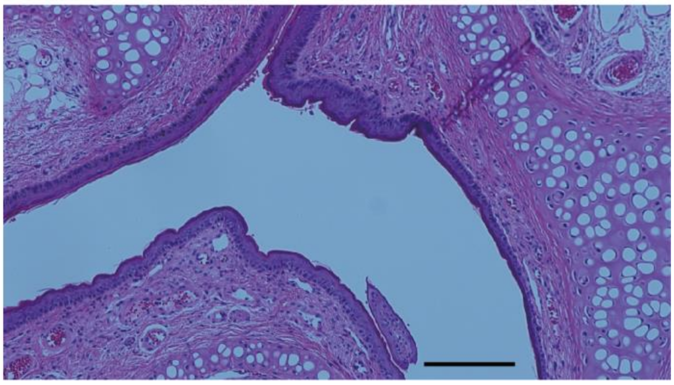

Supplementary Figure 15. Effect of PIAS on rat nasal tissue. Animals colonised by methicillin-resistant *Staphylococcus aureus* JKmrSA1 were subjected to PIAS using SA–IR700 conjugate. Histological analysis was performed on untreated (**a**) and PIAS-treated (**b**) nasal tissues. PIAS treatment did not induce any changes against irradiated nasal tissues. PIAS conditions: SA–IR700 conjugate, 5 µg/animal; near-infrared light illumination, 50 J/cm^2. SA–IR700 was used in both the PIAS (−) and (+) groups. Scale bars indicate 200 µm.

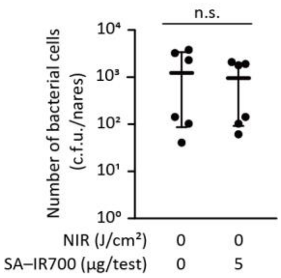

Supplementary Figure 16. Effect of SA–IR700 conjugate on MRSA of rat. Animals colonised by methicillin-resistant *Staphylococcus aureus* JKmrSA1 were treated with SA–IR700 conjugate. Subsequently, the samples were cultured on mannitol salt agar containing egg yolks. c.f.u., colony-forming units; NIR, near-infrared light; n.s., not significant ($p > 0.05$). Median and IQR values are shown. A two-tailed unpaired Student's t-test .

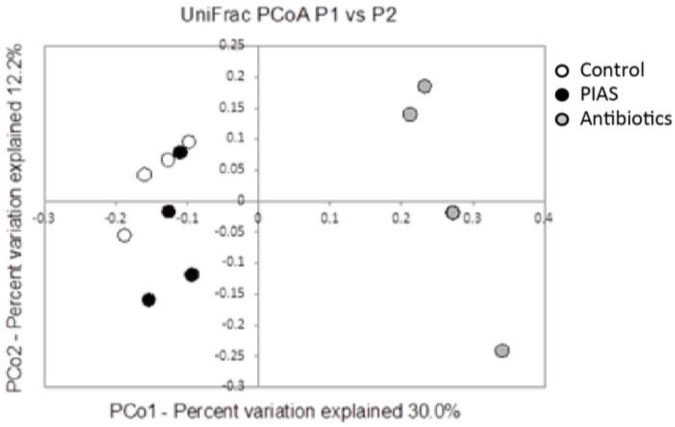

Permanova

Category	R^2	*p* value
Control vs. PIAS	0.13587	0.5754
Control vs. antibiotics	0.36482	0.02697

Supplementary Figure 17. Principal coordinate analysis (PCoA) of UniFrac distances of 16S rRNA genes. PCoA was performed on an unweighted UniFrac distance to compare the test samples using gene sequence data[5]. Control, phosphate-buffered saline, antibiotics, vancomycin, and rifampicin.

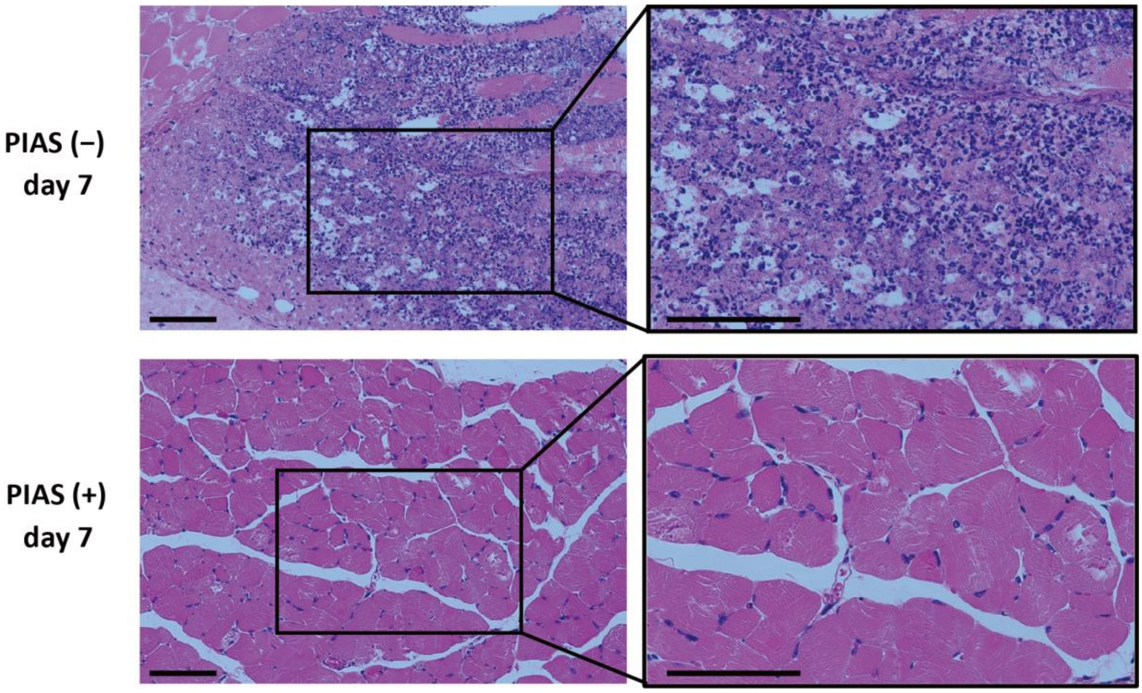

Supplementary Figure 18. Histochemical analysis of methicillin-resistant *Staphylococcus aureus*-infected thigh tissues treated with and without PIAS. At day 7 after infection, haematoxylin and eosin staining was performed on the thigh tissues. Massive inflammatory cell infiltration was observed in the untreated control sample (upper images) compared to the PIAS-treated sample (lower images). Low-compression images are shown. Scale bars indicate 100 µm.

Supplementary References

1 Usui, A., Murai, M., Seki, K., Sakurada, J. & Masuda, S. Intracellular localization of *Staphylococcus aureus* within primary cultured mouse kidney cells. *Microbiol*. *Immunol*. **36,** 545−550 (1992).

2 Lehar, S. M. et al. Novel antibody-antibiotic conjugate eliminates intracellular *S. aureus*. *Nature* **527,** 323−328 (2015).

3 Christensen, G. D., Baldassarri, L. & Simpson, W. A. Methods for studying microbial colonization of plastics. *Methods Enzymol*. **253,** 477−500 (1995).

4 Stepanović, S. et al. Quantification of biofilm in microtiter plates: overview of testing conditions and practical recommendations for assessment of biofilm production by staphylococci. *APMIS* **115,** 891−899 (2007).

5 Lozupone, C., Lladser, M. E., Knights, D., Stombaugh, J. & Knight, R. UniFrac: an effective distance metric for microbial community comparison. *ISME J*. **5,** 169−172 (2011).
