# Supplementary material for: Antimicrobial strategy for targeted elimination of different microbes, including bacterial, fungal and viral pathogens
Source: Commun Biol. 2022 Jul 4;5:647. doi: 10.1038/s42003-022-03586-4 (PMC9253063; doi:10.1038/s42003-022-03586-4)
Supplement: Supplementary file 2 — Description of Additional Supplementary Files [file 42003_2022_3586_MOESM2_ESM.pdf]

# Description of Additional Supplementary Files

**File name:** Supplementary Data 1
**Description:** Source data underlying bar graphs in Fig 2b-e, 3-4, and plots in 5a-b, 5d-f.
