# Supplementary material for: Antimicrobial strategy for targeted elimination of different microbes, including bacterial, fungal and viral pathogens
Source: Commun Biol. 2022 Jul 4;5:647. doi: 10.1038/s42003-022-03586-4 (PMC9253063; doi:10.1038/s42003-022-03586-4)
Supplement: Supplementary file 4 — Reporting Summary [file 42003_2022_3586_MOESM4_ESM.pdf]

nature research

Corresponding author(s): Makoto Mitsunaga and Tadayuki iwase

Last updated by author(s): Apr 5, 2022

Reporting Summary

Nature Research wishes to improve the reproducibility of the work that we publish. This form provides structure for consistency and transparency in reporting. For further information on Nature Research policies, see our Editorial Policies and the Editorial Policy Checklist.

Statistics

For all statistical analyses, confirm that the following items are present in the figure legend, table legend, main text, or Methods section.

n/a	Confirmed	
☐	☒	The exact sample size (n) for each experimental group/condition, given as a discrete number and unit of measurement
☐	☒	A statement on whether measurements were taken from distinct samples or whether the same sample was measured repeatedly
☐	☒	The statistical test(s) used AND whether they are one- or two-sided<br>*Only common tests should be described solely by name; describe more complex techniques in the Methods section.*
☐	☒	A description of all covariates tested
☐	☒	A description of any assumptions or corrections, such as tests of normality and adjustment for multiple comparisons
☐	☒	A full description of the statistical parameters including central tendency (e.g. means) or other basic estimates (e.g. regression coefficient) AND variation (e.g. standard deviation) or associated estimates of uncertainty (e.g. confidence intervals)
☐	☒	For null hypothesis testing, the test statistic (e.g. F, t, r) with confidence intervals, effect sizes, degrees of freedom and P value noted<br>*Give P values as exact values whenever suitable.*
☒	☐	For Bayesian analysis, information on the choice of priors and Markov chain Monte Carlo settings
☒	☐	For hierarchical and complex designs, identification of the appropriate level for tests and full reporting of outcomes
☒	☐	Estimates of effect sizes (e.g. Cohen's d, Pearson's r), indicating how they were calculated

Our web collection on statistics for biologists contains articles on many of the points above.

Software and code

Policy information about availability of computer code

Data collection	N/A
Data analysis	N/A

For manuscripts utilizing custom algorithms or software that are central to the research but not yet described in published literature, software must be made available to editors and reviewers. We strongly encourage code deposition in a community repository (e.g. GitHub). See the Nature Research guidelines for submitting code & software for further information.

Data

Policy information about availability of data

All manuscripts must include a data availability statement. This statement should provide the following information, where applicable:

- Accession codes, unique identifiers, or web links for publicly available datasets
- A list of figures that have associated raw data
- A description of any restrictions on data availability

The data that support the findings of this study are available from the corresponding author upon reasonable request.

Field-specific reporting

Please select the one below that is the best fit for your research. If you are not sure, read the appropriate sections before making your selection.

☒ Life sciences ☐ Behavioural & social sciences ☐ Ecological, evolutionary & environmental sciences

For a reference copy of the document with all sections, see nature.com/documents/nr-reporting-summary-flat.pdf

Life sciences study design

All studies must disclose on these points even when the disclosure is negative.

Sample size	We first conducted experiments using a small number of experimental animals (at least 3 mice per group). Results obtained these experiments were considered to be statistically significant at a P value < 0.05. To ensure robust statistical rigour, we performed additional in vivo experiments. The additional experiments indicated that aPIT produces reproducible results. Finally, Mice (n = 8) were used in almost all experiments in the study.
Data exclusions	No data were excluded from the analyses.
Replication	Almost all experiments in the study were performed at least thrice to confirm reproducibility.
Randomization	Control groups were designed in all experiments in the study; there are no human participants and clinical studies in the study.
Blinding	There are no human participants and clinical studies in the study.

Reporting for specific materials, systems and methods

We require information from authors about some types of materials, experimental systems and methods used in many studies. Here, indicate whether each material, system or method listed is relevant to your study. If you are not sure if a list item applies to your research, read the appropriate section before selecting a response.

Materials & experimental systems

n/a	Involved in the study	
☐	☒	Antibodies
☐	☒	Eukaryotic cell lines
☒	☐	Palaeontology and archaeology
☐	☒	Animals and other organisms
☒	☐	Human research participants
☒	☐	Clinical data
☒	☐	Dual use research of concern

Methods

n/a	Involved in the study	
☒	☐	ChIP-seq
☐	☒	Flow cytometry
☒	☐	MRI-based neuroimaging

Antibodies

Antibodies used	Anti-S. aureus monoclonal antibody (mAb) (SA; clone Staph12-569.3, murine IgG3) was purchased from QED Bioscience Inc. (San Diego, CA, USA). Anti-C. albicans mAb (CA; clone MC3, murine IgG3) was purchased from ISCA Diagnostics Ltd. (Exeter, UK). Anti-T7 phage mAb (T7•Tag Antibody, murine IgG2b) was purchased from Merck KGaA (Darmstadt, Germany). Anti-human epidermal growth factor receptor 2 mAb, trastuzumab (Herceptin, humanized IgG1) was purchased from Chugai Pharmaceutical Co. Ltd. (Tokyo, Japan).
Validation	The specificity of antibodies was evaluated prior to the use in experiments.

Eukaryotic cell lines

Policy information about cell lines

Cell line source(s)	The NIH/3T3 cells used in the study were purchased from ATCC (VA, USA)and have not yet authenticated since then.
Authentication	None.
Mycoplasma contamination	We have routinely tested mycoplasma contamination by using MycoAlert (Lonza, ME, USA) and confirmed that cells were negative for contamination.
Commonly misidentified lines (See ICLAC register)	N/A

Animals and other organisms

Policy information about studies involving animals; ARRIVE guidelines recommended for reporting animal research

Laboratory animals	Six- to ten-week-old cotton rats (Sigmodon hispidus) were obtained from the Animal Research Center, University of Occupational and Environmental Health School of Medicine (Fukuoka, Japan). Five- to seven-week-old female BALB/c mice were obtained from Clea Japan (Tokyo, Japan).
Wild animals	The study did not involved wild animals.
Field-collected samples	The study did not involve samples collected from the field.
Ethics oversight	Animal studies were performed in accordance with the guidelines established by the Animal Care Committee at the Jikei University School of Medicine.

Note that full information on the approval of the study protocol must also be provided in the manuscript.

Flow Cytometry

Plots

Confirm that:

☐ The axis labels state the marker and fluorochrome used (e.g. CD4-FITC).

☐ The axis scales are clearly visible. Include numbers along axes only for bottom left plot of group (a 'group' is an analysis of identical markers).

☐ All plots are contour plots with outliers or pseudocolor plots.

☐ A numerical value for number of cells or percentage (with statistics) is provided.

Methodology

Sample preparation	Sample prep; mAb–IR700 conjugate (1 μg) was added to approximately 1 × 10^5 colony-forming units (c.f.u.) of bacterial suspension (total volume of 100 μl) and incubated 1 h at 4 °C followed by cell washing with RPMI.
Instrument	MACSQant analyzer; Miltenyi Biotec
Software	cells were analyzed with MACSQant analyzer
Cell population abundance	N/A
Gating strategy	N/A

☐ Tick this box to confirm that a figure exemplifying the gating strategy is provided in the Supplementary Information.
